# Suicide in adults within 30 days of discharge from hospital emergency department

**DOI:** 10.1007/s11739-025-03921-7

**Published:** 2025-04-25

**Authors:** Tamara Maestre-Orozco, Vicente Soriano, Dolores-Elvira Sánchez-Gómez, Begoña Espinosa, Hilario Blasco-Fontecilla, Lucía Gallego, Pere Llorens, Jose-Manuel Ramos-Rincon

**Affiliations:** 1 Department of Internal Medicine, Dr. Balmis General University Hospital, Alicante Institute for Health and Biomedical Research (ISABIAL), Alicante, Spain; 2 UNIR Health Sciences School and Medical Center, Madrid, Spain; 3 Department of Emergency Medicine, Hospital Comarcal del Noroeste, Caravaca de la Cruz, Murcia, Spain; 4 Department of Emergency Medicine, Dr. Balmis General University Hospital, Alicante Institute for Health and Biomedical Research (ISABIAL), Alicante, Spain; 5 Emooti Policlinic, Madrid, Spain; 6 Center of Biomedical Network Research on Mental Health (CIBERSAM), Madrid, Spain; 7 Department of Clinical Medicine, University Miguel Hernández of Elche, Carretera 332 s/n, 03550 Sant Joan d’Alacant, Alicante, Spain

**Keywords:** Suicide, Emergency, Mental disorders, Suicide predictors

## Abstract

Suicide remains a frequent cause of death in adults, often overlooked despite known risk factors like mental disorders, older age, and chronic illness. Emergency department visits offer critical opportunities to identify suicidal ideation and provide preventive care. This study reviewed deaths within 30 days of discharge from the emergency department of a major public hospital in Alicante, Spain, focusing on deaths by suicide. Over five years, 529,312 emergency visits were recorded, with 453,599 patients (85.7%) discharged home. Of the 356 patients (0.078%) who died within 30 days post-discharge, 7 (1.97%) died by suicide. Among these, four had a psychiatric history, and one had a history of substance abuse, both established risk factors. The overall suicide rate among adults discharged from the emergency department was 1.54 per 100,000. Compared with those who died from other causes within 30 days of discharge, patients who died by suicide were younger (median age 72 vs. 83 years; p = 0.037, adjusted OR: 0.59, 95% CI: 0.34–1.03; p = 0.06), had a lower Charlson Comorbidity Index (median 3 vs. 6; p = 0.003), and were more often male (6/7 vs. 50.1%; p = 0.1). The presence of psychiatric history was the most significant risk factor for suicide within 30 days of discharge from the emergency department among patients aged 15 and older. Men were more likely than women to die by suicide, with the majority between the ages of 65 and 79. Notably, only one individual had a prior suicide attempt.

## Introduction

Around 800,000 people die by suicide worldwide every year, accounting for 1.5% of all deaths [1]. Suicide ranks among the top ten leading causes of death in Western countries and is the leading cause of death among individuals aged 15 to 24 years [2]. In response, international experts and the WHO have called for urgent interventions [3, 4].Recognizing predisposing factors is crucial for implementing preventive strategies. Mood disorders and substance use behaviors are among the most significant predictors of suicide [2]. However, suicide research faces a paradox: although a previous suicide attempt is the most relevant risk factor for suicide [5], most suicides occur on the first or second attempt, which limits predictive capabilities [6– 9] Additionally, precipitating factors, such as stressful life events, may particularly affect vulnerable individuals, aligning with the diathesis-stress model of suicide [10, 11].

While a prior suicide attempt is a significant risk factor, most individuals who die by suicide do so on their first attempt, complicating prevention strategies. For instance, a previous study found that 60.4% of suicides occurred on the first attempt and 92.3% on either the first or second attempt [6]. Close monitoring and proper management by psychiatrists could significantly benefit these patients. However, the majority of those who die by suicide are not under the care of mental health professionals but are instead treated in primary care facilities [12, 13]. Identifying suicide ideation or behaviors in individuals visiting emergency departments (ED) for any reason could provide an alternative means of reaching this at-risk population. However, data on suicide risk profiles within ED settings is scarce [14].

In Spain, suicides increased from 3,539 in 2018 to 4,003 in 2021, the latest year for which data is available [15]. In a prior study, we reported that nearly 90% of individuals who died by suicide had not been assessed previously in an ED, despite one in four having a history of prior suicide attempts. Among those whose previous suicide attempt was evaluated in an ED, the most prevalent risk factors included alcohol use disorder and older age, with 87% aged between 40 and 59 years [13].

In this study, we report the results of a retrospective analysis conducted on all individuals seen consecutively in the ED of a large tertiary public hospital in Alicante, Spain, over five years. Those who died within 30 days of hospital discharge were identified, and the causes of death were examined. Our main objective was to identify suicide risk factors in patients attending the ED. Patients who died by suicide were identified and compared with the remaining cohort."

## Methods

### Study design

This was an observational, cross-sectional, and retrospective study examining all individuals who visited the adult ED of Hospital Dr. Balmis in Alicante between January 1, 2017, and December 31, 2021. This hospital is a large, tertiary public healthcare institution. Only individuals aged 14 years and older were included in the adult ED analysis. In a second step, only those who were discharged directly home were selected, excluding patients admitted to the hospital or transferred to other clinics.

Deaths occurring within 30 days post-discharge were identified using information from the *Subdirección General de Epidemiología de la Dirección General de Salud Pública y Adicciones* [16]. This public organization is responsible for planning, directing, coordinating, and supervising the administrative units under its authority. Each death certificate was reviewed individually to confirm the cause of death. Suicide as the cause of death was deemed definitive or highly probable by two researchers (TMO and DESG), with a third researcher (PL) consulted in cases of disagreement.

Using the administrative platform *Transit of the Health Department of the Regional Government*, we collected further information on key demographic and social parameters, including sex, age, cause of death, date and diagnosis of the last ED visit, total number and diagnoses of ED visits, days from the last ED visit to death, total number of prior primary care consultations, comorbidities and Charlson Comorbidity Index (CCI), psychiatric diagnoses, admissions to psychiatric units, outpatient follow-up by psychiatrists, history of suicide attempts, substance use, and functional and cognitive status."

### Statistical analyses

Quantitative variables were reported as median and interquartile ranges. Qualitative variables did not follow a normal distribution, as confirmed by the Kolmogorov-Smirnov test. Categorical variables are expressed as counts and percentages.

Differences between the two groups (deaths due to suicide versus other causes) for quantitative variables were assessed using the Mann-Whitney U test. Qualitative data were compared using the Chi-square test, with Fisher's correction applied when necessary. The effect size was estimated using odds ratios (OR) with 95% confidence intervals. To identify independent predictors of suicide, we conducted a multivariable logistic regression analysis. Variables with p-values below 0.2 in the univariate analysis were entered into the multivariable logistic regression model using a stepwise selection method with the likelihood ratio test. Statistical analyses were performed using SPSS software version 28.0 (IBM, Armonk, USA).

### Legal and ethics

The study was approved by the Clinical Research Ethics Committee of the Dr. Balmis General Hospital, Alicante (ref. PI2023-125). Research was conducted in accordance with the Helsinki Declaration as revised in 1989.

## Results

### Epidemiological findings

During the study period, 529,312 visits to the hospital ED were recorded for adults (aged >14 years), of which 453,599 (85.7%) were discharged home after appropriate assessment and care. Within 30 days post-discharge, 356 (0.078%) of these patients died, including 7 patients who died by suicide. Thus, the overall suicide rate per 100,000 patients discharged from the hospital ED was 1.54. The annual ED visits, discharges home from the ED, patients who died at home within the first 30 days, and patients who died at home by suicide are shown in Table 1.

**Table 1:** The annual ED visits, discharges home, patients who died at home within the first 30 days, and due to suicide

	2017	2017	2019	2020º	2021	Total
ED visit, N	108,718	109,298	113,724	86,713	100,859	519,312
ED discharges home, N	95,759	95,823	99,831	74,418	87,768	453,599
Death at home within 30 days, N (%)*	68 (0.07)	81(0.08)	67 (0.07)	64 (0.9)	76 (0.09)	356 (0.08)
Death at home due to suicide, N (%)**	3 (4.41)	0 (0.0)	0 (0.0)	2 (3.13)	2 (2.63)	7 (1.97)

^*^Percentage = Death at home within 30 days/ ED discharges home x 100

^**^Percentage = Death at home due to suicide / Death at home within 30 days x 100

### Deaths due to suicide compared to other causes

When analyzing sex, age, days post-discharge, and comorbidities in all patients who died within 30 days (Table 2), it was observed that individuals who died by suicide, compared to others, were younger (median age 72 vs. 83 years; p = 0.037) and had a lower comorbidity index (median CCI score: 3 vs. 6; p = 0.003). Additionally, most suicides occurred in males (6 out of 7), while other deaths were equally distributed between sexes. Finally, the time between hospital discharge and death was shorter for those who died by suicide than for other deaths (median of 3 vs. 10 days). In the multivariate analysis, the adjusted OR for comorbidity index was 0.59 (95% CI: 0.34–1.03; p = 0.06), for male sex it was 6.06 (95% CI: 0.70–52; p = 0.10), and for age it was 0.85 (95% CI: 0.93–1.09; p = 0.8).

**Table 2:** Comparison of deaths due to suicide and other causes

Variables	Suicide (n = 7)	Other causes of death (n = 349)	P	OR (CI 95%)
Sex				
Men	6 (85.7)	175 (50.1)	0.100	5.96 (0.71–50.1)
Women	1 (14.3)	174 (49.9)		Ref
Age (years)				
Median (IQR)	72 (61.5–76)	83 (72–88)	0.037	0.95 (0.91–0.99)
18-64	2 (28.6)	49 (14.0)	Ref	Ref.
65-79	3 (42.9)	87 (24.9)	0.856	0.84 (0.13–5.32)
≥ 80	2 (28.6)	213 (61.0)	0.147	0.23 (0.03–1.67)
Comorbidities. Charlson index				
Median (IQR)	3 (2–6)	6 (5–8)	0.003	0.61 (0.44–0.84)
≤ 6	6 (85.7)	186 (53.3)	0.129	Ref.
≥ 7	1 (14.3)	163 (46.7)		0.19 (0.02–1.59)
Days after discharge				
Median (IQR)	3 (2–14)	10 (4–18)	0.360	0.95 (0.86–1.56)
≤ 7	4 (57.6)	144 (41.3)	0.456	Ref.
≥ 8	3 (42.9)	205 (58.7)		0.52 (0.11–2.39)

### Main features of patients who died due to suicide

Most patients who died by suicide were men (85.7%) and aged between 65 and 79 years. All were active, independent, and had no cognitive impairments, except one (case 6) who had mild memory loss.

The most common method of suicide was jumping, reported in four cases (57.1%). Other methods included beheading, hanging, and poisoning. Only one individual had a prior suicide attempt. However, more than half (n = 4; 57.1%) had a psychiatric history and were under psychiatric follow-up, though none had required psychiatric hospitalization. Most individuals had been recently diagnosed with anxiety and/or severe depression. Only one had a history of substance abuse (case 5). Two individuals did not have any prior mental disorder diagnosis (cases 3 and 4).

At their last ED visit, two of the seven patients who ultimately died by suicide within 30 days post-discharge had received a psychiatric diagnosis or documented suicidal ideation/behavior. Four of these patients died by suicide within the first three days after the ED visit. Additionally, four had visited a general practitioner (GP) shortly before going to the ED, presenting similar symptoms (e.g., abdominal pain, dysphagia). Table 3 summarizes the main clinical features of the patients who died by suicide

**Table 3:** Main features of patients who commited suicide

Patient	Age (years-old)	Sex	Suicide method	Last diagnosis at ED*	Prior hospital visits		Days since hospital discharge	Prior psychiatric history	Prior suicide attempts	Prior psychiatric consultation	Psichiatric treatment	Posterior ED psychiatric consultation
					ED*	GP**						
1	80	male	Precipitation	Abdominal pain	2	1	3	Depression	0	Yes	No	No
2	87	male	Precipitation	Abdominal pain	2	0	1	Anxiety	0	No	No	No
3	69	male	Beheaded	Refractory pain	1	2	23	No	0	No	No	No
4	72	male	Hanging	Dysphagia	2	2	17	No	0	No	No	No
5	65	male	Opioid overdose	Drug intoxication	2	1	1	Anxiety	0	Yes	Yes	No
6	74	male	Jumping	Behavior disorder	3	4	3	Anxiety and depression	2	Yes	Yes	No
7	47	female	Jumping	Dysphagia and fatigue	2	>10	11	Anxiety and depression	0	Yes	Yes	No

^*^ED. emergency department

^**^GP. general practitioner

## Discussion

This observational study identified several risk factors for suicide among adults who died by suicide within 30 days after being discharged from the ED. Men were more likely to die by suicide than women, particularly those aged 65 to 79 years. Additionally, patients who died by suicide tended to do so shortly after their initial ED visit; notably, four of the seven suicides occurred within the first three days post-discharge. Adults who died by suicide were generally younger and had a lower Charlson Comorbidity Index (CCI) compared to those who died from other causes. Despite more than half having mental disorders and being under psychiatric care, only one had a prior suicide attempt. Additionally, one individual presented to the ED for drug intoxication and later died by suicide through opioid overdose.

The sociodemographic profile of suicide risk in our study (older males) aligns with findings from a study conducted in California [17]. In that study, the authors analyzed the incidence of suicide or other causes of death among patients who visited the ED for self-harm or suicidal ideation. They observed higher suicide rates among men and individuals aged 65 years and older. Furthermore, patients presenting with self-harm or suicidal thoughts were at increased risk of suicide after ED discharge. The authors emphasized the importance of comprehensive mental health follow-up for this population upon discharge, underscoring the critical role EDs can play in detecting and preventing suicide risk

Several of our findings underscore the impact of physical illnesses on individuals who die by suicide. First, although the suicide group had a lower CCI compared to those who died from other causes, they still had a median of three somatic comorbidities, indicating compromised physical health. Second, while most individuals who died by suicide had mental disorders, only one had a prior suicide attempt. Interestingly, most of these patients did not visit the ED for psychiatric reasons but for somatic complaints and had previously consulted their GP for the same issues. Abdominal pain and dysphagia were the primary discharge diagnoses from the ED. In one case, inadequate pain management in a cancer patient was noted. Only two of the seven individuals who died by suicide were discharged from the ED with a psychiatric diagnosis.

Overall, our findings suggest that medical conditions may play a more dominant role than mental disorders in those who die by suicide after ED visits. Numerous studies have highlighted the significance of chronic serious illnesses, such as cancer, respiratory failure, or advanced HIV infection, as predictors of suicide [2, 18]. Additional risk factors include male gender, somatic diseases, and alcohol abuse [6, 19].

Individuals with psychiatric disorders are at heightened risk for suicide [2, 7, 9]. Anxiety and depression were the most common psychiatric comorbidities among our patients who died by suicide. However, somatic symptoms may obscure the underlying psychological distress in these patients. In fact, most individuals in our study who died by suicide sought care at the ED for physical rather than mental health complaints [20]. Unfortunately, treating physicians often overlooked these patients as being at high risk for suicide, a finding echoed in another research [20, 21].

Regarding suicide methods, Crandall et al. [20] reported a higher suicide rate among men, with firearms being the most common method in the United States due to easier access to weapons. In contrast, in our study in Spain, the predominant method was jumping from a height, a finding also observed in previous Spanish research [13]. This difference is significant, as access to methods is a crucial aspect of suicide prevention [22].

One of the most important findings from our study is the low recognition of suicide risk in ED patients, as most suicides occurred shortly after discharge. A study from Ethiopia [18] found that suicidal behaviors were more prevalent among ED patients than in the general population. Similarly, research from New Mexico [20] reported a higher suicide rate among patients discharged from the ED compared to population-based estimates. Patients presenting with suicidal ideation, drug overdoses, or self-harm injuries were particularly at risk for suicide post-discharge, underscoring the need for thorough psychiatric follow-up. As in our study, most ED patients who later died by suicide had not been identified as at-risk during their visits.

Visiting the ED for any reason is not typically viewed as a suicide risk factor. However, all patients who died by suicide in our study had multiple visits to the ED and/or GP clinics in the weeks or months before their deaths. This finding aligns with other studies; for instance, a UK study [14] noted that 39% of individuals who died by suicide had visited an ED in the year before their death, with 61% of these visits being for reasons other than self-harm. Additionally, a Texas study on mental health in ED [23] found that 11.6% of patients had suicidal thoughts, and 2% were actively planning suicide. Subsequent record reviews revealed that 25 of the 31 patients with suicide plans went undetected during their ED visit; four of these individuals attempted suicide within 45 days of discharge. This highlights the frequent oversight of suicide risk in ED assessments and suggests that recognizing patterns of frequent prior visits could improve risk detection [24].

Our findings highlight the inadequate clinical detection of suicide risk in patients who visit the ED with symptoms that appear unrelated to suicide, a concern echoed in previous studies [14]. Furthermore, most suicides occur among individuals with no history of prior attempts. Specifically, over 60% and 90% of individuals who die by suicide do so on their first or second attempt, respectively [12]. Additionally, most people who die by suicide are typically seen by primary healthcare services rather than mental health services [12, 13].

Finally, we would like to stress a very relevant question: should all patients visiting ED be psychiatrically examined? Would this measure contribute to reduce suicide risk? We understand that this is a very tricky question that surpasses the objective of the current study. However, if we decide to response “yes” to this question, the problem would be how to do it given the characteristic of ED. A possible solution to make this a reality would be the implementation of brief protocols including very brief scales capable of identifying those at higher suicide risk.

For instance, the short Personality Life Event (S-PLE) scale, a brief scale of just 6 items and less than one minute of administration, fairly discriminated between medical controls, psychiatric controls and suicide attempters. The S-PLE scale discriminated between past non-suicide attempters and past suicide attempters with sensitivity of 80%, specificity of 75%, and area under the ROC curve of 88%. The S-PLE is composed by six items, being the most discriminative items ‘‘I often feel empty inside’’ and ‘‘Adult self-harm’’. In addition to the short time employed (less than one minute), another key advantage of the S-PLE scale is its potential to indirectly assess suicide risk. The items on the S-PLE scale evaluate how the individual perceives themselves and their relationships with their social, occupational, and emotional environments. This is crucial, as many individuals who attempt or complete suicide do not express suicidal thoughts in prior appointments with their healthcare providers [25]. This lack of communication may stem from fears of stigma, hospitalization, or disruption of their plans [25, 26]. In this context, the S-PLE is particularly valuable; aside from one item on adult self-harm, it contains no direct questions about suicide attempts. This makes the S-PLE especially useful for patients who might otherwise withhold such information, allowing clinicians to assess suicide risk without relying solely on explicit reports of suicidal ideation. Yet, the is no evidence that the S-PLE (or any other scale) has enough discriminative capability to detect not only suicide attempters, but suicide completers.

Our study has several limitations. First, it shares the typical constraints of cross-sectional, retrospective studies, such as selection biases, which may have affected the accuracy of our findings. Second, unlike other studies investigating patients who die by suicide post-discharge, our sample was highly selective, as we included only those patients who were discharged directly to their homes. Therefore, our results cannot be generalized to the overall population of ED patients who are discharged, or to the general population. Third, the sample size of suicide cases was very small (n = 7). Fourth, we could include only patients aged 14 years or older because the research was conducted in adult EDs, which serve patients over 14 years old; pediatric EDs, which attend to those under 15, are located separately. Finally, we lacked information on some potentially relevant risk factors, such as the “chief complaint/reason for admission”.

In summary, our study reveals crucial insights into the risk factors associated with suicide among adults within 30 days of discharge from the ED. Our findings underscore a demographic profile of older males, often presenting with somatic rather than psychiatric complaints. Despite frequent ED or GP visits prior to their deaths, most patients were not identified as being at suicide risk. This suggests that medical comorbidities and physical health issues may obscure underlying mental health concerns, thereby complicating risk assessments. Our observations align with prior research, which highlights the role of chronic illnesses, male gender, and somatic complaints in suicide risk, especially when there is no prior suicide attempt or explicit psychiatric diagnosis at discharge. The study emphasizes that a significant proportion of suicides happen shortly after ED discharge, often without the individuals being flagged as at risk during their visits. The method of suicide observed in Spain differs from patterns seen in the United States, where firearm access is more prevalent, suggesting that local context and method accessibility play important roles in preventive strategies.

Ultimately, this study points to a need for improved suicide risk detection among patients presenting with somatic symptoms and for proactive mental health follow-ups post-discharge. There is a pressing need for a more thorough assessment of suicide risk in patients visiting ED, particularly those with mental disorders such as mood and anxiety disorders [14]. Given that predicting suicide can be very difficult, efforts should focus more on prevention, strengthening the collaboration between ED and mental health services, as many suicides appear to be preceded by ED visits, even for reasons that seem unrelated. Recognizing repeated ED or primary care visits as potential indicators could enhance early detection and intervention, potentially saving lives.

## References

[1] Naghavi M (2019) Global, regional, and national burden of suicide mortality 1990 to 2016: systematic analysis for the global burden of disease study 2016. BMJ 364:l94 31339847 10.1136/bmj.l94 PMC6598639

[2] Fazel S, Runeson B (2020) Suicide. N Engl J Med. 382:266–274 31940700 10.1056/NEJMra1902944 PMC7116087

[3] Melhem N, Moutier C, Brent D (2023) Implementing evidence-based suicide prevention strategies for greatest impact. Focus. Am Psychiatr Publ. 21:117–128 37201145 10.1176/appi.focus.20220078 PMC10172552

[4] World health organization: WHO. suicide [Internet]. 2023. Available at: http://www.who.int/es/news-room/fact-sheets/detail/suicide

[5] Oquendo MA, Currier D, Mann JJ (2006) Prospective studies of suicidal behavior in major depressive and bipolar disorders: what is the evidence for predictive risk factors? Acta Psychiatr Scand. 114(3):151–158 16889585 10.1111/j.1600-0447.2006.00829.x

[6] Parra-Uribe I, Blasco-Fontecilla H, García-Parés G et al (2013) Attempted and completed suicide: not what we expected? J Affect Dis 150:840–6 23623420 10.1016/j.jad.2013.03.013

[7] Hoven C, Mandell D, Bertolote JM (2010) Prevention of mental ill-health and suicide: public health perspectives. Eur Psychiatry. 25:252–6 20452753 10.1016/j.eurpsy.2010.01.011

[8] Isometsä ET (2001) Psychological autopsy studies - a review. Eur Psychiatry. 16:379–385 11728849 10.1016/s0924-9338(01)00594-6

[9] Arsenault-Lapierre G, Kim C, Turecki G (2004) Psychiatric diagnoses in 3275 suicides: a meta-analysis. BMC Psychiatry. 4:37 15527502 10.1186/1471-244X-4-37 PMC534107

[10] Mann JJ, Waternaux C, Haas GL, Malone KM (1999) Toward a clinical model of suicidal behavior in psychiatric patients. Am J Psychiatry. 156(2):181–189 9989552 10.1176/ajp.156.2.181

[11] Oquendo MA, Sullivan GM, Sudol K et al (2014) Toward a biosignature for suicide. Am J Psychiatry. 171(12):1259–1277 25263730 10.1176/appi.ajp.2014.14020194 PMC4356635

[12] Artieda-Urrutia P, Parra-Uribe I, Garcia-Pares G, Palao D, de Leon J, Blasco-Fontecilla H (2014) Management of suicidal behaviour: is the world upside down? Aust N Z J Psychiatry. 48:399–401 24589981 10.1177/0004867414525847

[13] Parra-Uribe I, Blasco-Fontecilla H, Garcia-Parés G et al (2017) Risk of re-attempts and suicide death after a suicide attempt: a survival analysis. BMC Psychiatry. 17:163 28472923 10.1186/s12888-017-1317-z PMC5415954

[14] Gairin I, House A, Owens D (2003) Attendance at the accident and emergency department in the year before suicide: retrospective study. Br J Psychiatry. 183:28–33 12835240 10.1192/bjp.183.1.28

[15] Instituto Nacional de Estadística (INE). Estadística de defunciones según la causa de muerte año 2022. [Internet]. 2023. Available in: https://www.ine.es/prensa/edcm_2022.pdf

[16] Valenciana. Subdirección General de Epidemiología de la Dirección General de Salud Pública y Adicciones [Internet]. Valencia: Generalitat Valenciana; [cited 2025 Apr 12]. Available from: https://www.gva.es/es/inicio/atencion_ciudadano/buscadores/departamentos/detalle_departamentos?id_dept=24103 31834399 10.1001/jamanetworkopen.2019.17571 PMC6991205

[17] Goldman-Mellor S, Olfson M, Lidon-Moyano C, Schoenbaum M (2019) Association of suicide and other mortality with emergency department presentation. JAMA Netw Open. 2:e1917571 10.1001/jamanetworkopen.2019.17571 PMC6991205 31834399

[18] Wordefo D, Kassim F, Birhanu E, Mamo G (2023) Suicidal behaviors and associated factors among patients attending an emergency department: a facility-based cross-sectional study. BMC Psychiatry. 23:462 37357261 10.1186/s12888-023-04949-9 PMC10290805

[19] Giner L, Blasco-Fontecilla H, Perez-Rodriguez M et al (2013) Personality disorders and health problems distinguish suicide attempters from completers in a direct comparison. J Affect Disord. 151:474–483 23859005 10.1016/j.jad.2013.06.029

[20] Crandall C, Fullerton-Gleason L, Aguero R, LaValley J (2006) Subsequent suicide mortality among emergency department patients seen for suicidal behavior. Acad Emerg Med. 13:435–42 16531601 10.1197/j.aem.2005.11.072

[21] Maestre-Orozco T, Ramos-Rincón JM, Espinosa B et al (2024) Mortalidad tras el alta desde el servicio de urgencias hospitalario: análisis tras 453 599 episodios. Emergencias. 36:168–178 38818982 10.55633/s3me/018.2024

[22] Berman AL, Athey A, Nestadt P (2022) Effectiveness of restricting access to a suicide jump site: a test of the method substitution hypothesis. Inj Prev. 28:90–2 34417196 10.1136/injuryprev-2021-044240

[23] Claassen C, Larkin G (2005) Occult suicidality in an emergency department population. Br J Psychiatry 186:352–3 15802695 10.1192/bjp.186.4.352

[24] Gunnarsdottir O, Rafnsson V (2008) Death within 8 days after discharge to home from the emergency department. Eur J Public Health. 18:522–6 18550568 10.1093/eurpub/ckn045

[25] Smith EG, Kim HM, Ganoczy D, Stano C, Pfeiffer PN, Valenstein M (2013) Suicide risk assessment received prior to suicide death by veterans health administration patients with a history of depression. J Clin Psychiatry. 74:226–32 23561227 10.4088/JCP.12m07853 PMC4055158

[26] Isometsa ET, Heikkinen ME, Marttunen MJ, Henriksson MM, Aro HM, Lonnqvist JK (1995) The last appointment before suicide: is suicide intent communicated? Am J Psychiatry. 152:919–22 7755124 10.1176/ajp.152.6.919

